# Complete Genomic Sequence of the Thermophilic and Hydrogenotrophic Methanogen *Methanothermobacter* sp. Strain KEPCO-1

**DOI:** 10.1128/MRA.01034-19

**Published:** 2020-01-02

**Authors:** Byoung Seung Jeon, Hyunjin Kim, Young Wook Go, Young Gook Kim, Ji Sun Joo, Okkyoung Choi, Byoung-In Sang

**Affiliations:** a Department of Chemical Engineering, Hanyang University, Seoul, Republic of Korea; b Center for Applied Geosciences, University of Tübingen, Tübingen, Germany; c Korea Electric Power Research Institute, Daejeon, Republic of Korea; University of Southern California

## Abstract

Here, we describe the complete genome of *Methanothermobacter* sp. strain KEPCO-1, a thermophilic and hydrogenotrophic methanogen that was isolated from an anaerobic digester in Seoul, Republic of Korea. The genome of KEPCO-1 shares 96.98% of its sequence with Methanothermobacter marburgensis strain DSM 2133 and consists of 1,741,029 bp, with 1,822 protein-coding genes, 44 noncoding RNAs, and a GC content of 48.47%. The development of this genome will facilitate future genomic studies of KEPCO-1.

## ANNOUNCEMENT

Power-to-gas (P2G) is an electrical storage system (1) that has recently been considered as an approach to addressing load fluctuations associated with renewable energy (2, 3). Using this system, surplus electricity from renewable sources can be converted by water electrolysis into hydrogen, which can then be utilized in methane production with carbon dioxide by using the methanation process (3).

Here, we describe the isolation of *Methanothermobacter* sp. strain KEPCO-1 and the generation of its complete genome. KEPCO-1 grows optimally at 60°C with a pH of 7.0 to 7.5 and produces methane under pressurized conditions (approximately 150 to 200 kPa), using CO_2_ and H_2_ (4). The isolation process was conducted through sequential subculturing using general agar (4, 5). The strain was identified by 16S rRNA gene sequencing using the following primers: 40F (5′-GAT TAA GCC ATG CAA GTC GAA CGA-3′), 450F (5′-CTT CTG GAA TAA GGG CTG GGC A-3′), 765R (5′-CAT CGT TTA CGG CCA GGA CTA C-3′), and 1430R (5′-CTC CTC AAA GAA CCC AGA TTC GAC-3′). Once the strain was identified, whole-genome sequencing was performed.

KEPCO-1 was grown in basal medium at 60°C and 200 kPa for 7 days, and genomic DNA was extracted using the DNeasy UltraClean microbial kit (Qiagen Korea Ltd., Seoul, Republic of Korea) (6). The KEPCO-1 genome was analyzed using the PacBio RS II platform (PacBio single-molecule real-time sequencing) at Macrogen Co. Ltd. (Seoul, Republic of Korea), and the library was prepared using the SMRTbell template preparation kit (7). The raw data were assembled *de novo* by RS HGAP v3 and polished using Quiver v1 in SMRT Portal v2.3.0 (8).

During the preassembly step, filtering and assembly were conducted using preAssembler Filter v1 and preAssembler v2. Default parameters were used for all software unless otherwise specified. After the genome was assembled, gene functions were annotated via the NCBI Prokaryotic Genome Annotation Pipeline (https://www.ncbi.nlm.nih.gov/genome/annotation_prok).

The filtered data included a total of 1,099,392,337 bases. There were 81,220 postfiltered reads, encompassing 964,138,339 sequenced bases. The mean subread and *N*_50_ read lengths were 7,204 bases and 10,409 bases, respectively. The genome is composed of a single chromosome (1,741,029 bp), with an average reference coverage of 421×. One contig was generated and the two ends of the contig overlapped, which indicates that the genome is circular. Genome annotations identified a total of 1,866 genes, of which 1,822 were predicted to contain coding sequences, 37 were tRNAs, and 7 were rRNAs. The GC content was 48.75%.

The most closely related strain for KEPCO-1 was Methanothermobacter marburgensis DSM 2133 (9, 10), and the average nucleotide identity of KEPCO-1 with respect to the nucleotide sequence of DSM 2133 was 96.98% (11, 12). Furthermore, genes that were involved in energy production and conversion represented the largest proportion of genes in the KEPCO-1 genome (12). The number of genes and the proportion were 169 and 8.9%, respectively. This category is related to genes that are involved in the synthesis of enzymes, coenzymes, and prosthetic groups that are involved in CO_2_ and H_2_ utilization to produce methane (12).

### Data availability.

This whole-genome project (in GenBank format) and the corresponding raw files (in fastq format) have been deposited under GenBank accession numbers CP042937 and SRR10298064, respectively.
